# PLIP 2025: introducing protein–protein interactions to the protein–ligand interaction profiler

**DOI:** 10.1093/nar/gkaf361

**Published:** 2025-05-10

**Authors:** Philipp Schake, Sarah Naomi Bolz, Katja Linnemann, Michael Schroeder

**Affiliations:** Biotec and scads.ai, TU Dresden, 01307 Dresden, Germany; Biotec and scads.ai, TU Dresden, 01307 Dresden, Germany; Biotec and scads.ai, TU Dresden, 01307 Dresden, Germany; Biotec and scads.ai, TU Dresden, 01307 Dresden, Germany

## Abstract

PLIP, the protein–ligand interaction profiler, analyses molecular interactions in protein structures. PLIP detects eight types of non-covalent interactions. Initially focused on small-molecule, DNA, and RNA interactions to a protein, the current release incorporates protein–protein interactions. We document the usefulness of this feature by comparing PLIP interactions of the cancer drug venetoclax with the native protein–protein interaction of Bcl-2 and BAX. PLIP reveals how the drug mimics the native interaction, as there is critical overlap in the interaction profiles. PLIP is available as a web server, source code with containers, and Jupyter notebook. The PLIP web server is online at https://plip-tool.biotec.tu-dresden.de.

## Graphical Abstract

**Graphical Abstract:**
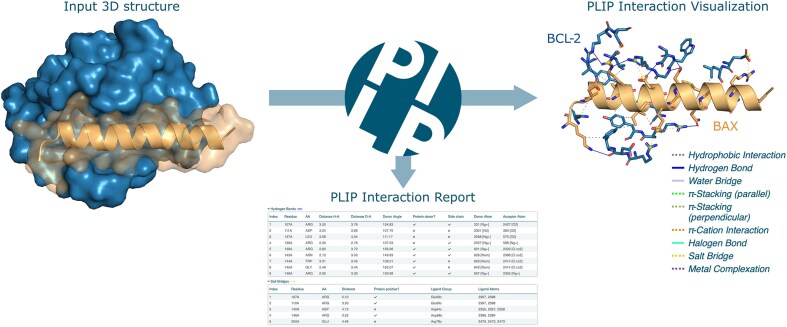

## Introduction

With the growth of the protein databank PDB [1] and the availability of millions of predicted protein structures [2, 3], there is a need to analyze molecular interactions between protein targets and small molecules, DNA, RNA, peptides, and proteins. PLIP, the protein ligand–interaction profiler, addresses this need by extracting eight types of interactions. Across all the protein–ligand interactions (PLIs) in the PDB, hydrogen bonds, hydrophobic contacts, water bridges, and salt bridges are the most abundant interactions with 37%, 28%, 11%, and 10%, followed by metal complexes, π-stacking, π-cation interactions, and halogen bonds at 9%, 3%, 1%, and 0.2%, respectively. Bolz *et al.* compared PLIs and protein–protein interactions (PPIs) and found that the most abundant interactions in PPIs match those found in PLIs, with the major difference being the absence of halogen bonds and metal complexations in PPIs. On average, a PLI has 12 non-covalent contacts, whereas a PPI has 48, consistent with the expectation that PPIs are generally larger [4].

Over the past decade, PLIP [5, 6] has been widely used in three main application areas:

1. Drug screening pipelines: PLIP can be adopted to prioritize candidates from large-scale docking experiments. To this end, Jang *et al.* used PLIP to reduce candidates of a COVID-19 docking screen by 90%. This reduction allowed the authors to experimentally verify seven of the final candidates. It turned out that the seven verified candidates share a common PLIP pattern [7].
2. Characterization of protein complexes: Chen *et al.* used PLIP to analyze molecular dynamics simulations of the S-adenosyl-L-methionine (SAM) riboswitch system. They observed that, despite minimal structural differences under varying conditions, the interaction patterns changed significantly, which directly correlated with the model’s free energy predictions [8].
3. Deep learning: Currently, there is a tremendous need for high-quality, well-curated datasets to benchmark machine learning approaches for drug-target prediction. Durairaj *et al.* recently published PLINDER [9], the largest and most annotated benchmark to date, comprising 449 383 PLIs. The authors used PLIP to identify protein residues interacting with small molecules, ensuring data integrity and preventing data leakage during the creation of training and testing splits.

## PLIP for protein–protein interactions

PLIP is well established for the analysis of small molecules, DNA, and RNA. However, recently, the structural characterization of PPIs gained prominence driven by two developments: on one hand, tools such as AlphaFold make large-scale PPI prediction widely and easily accessible, and on the other hand, drugs targeting PPIs begin to enter the market [10]. An example is venetoclax, which targets the interaction of B-cell lymphoma 2, Bcl-2, and the pro-apoptotic Bcl-2-associated X protein, BAX [11–13].

**Figure 1.**
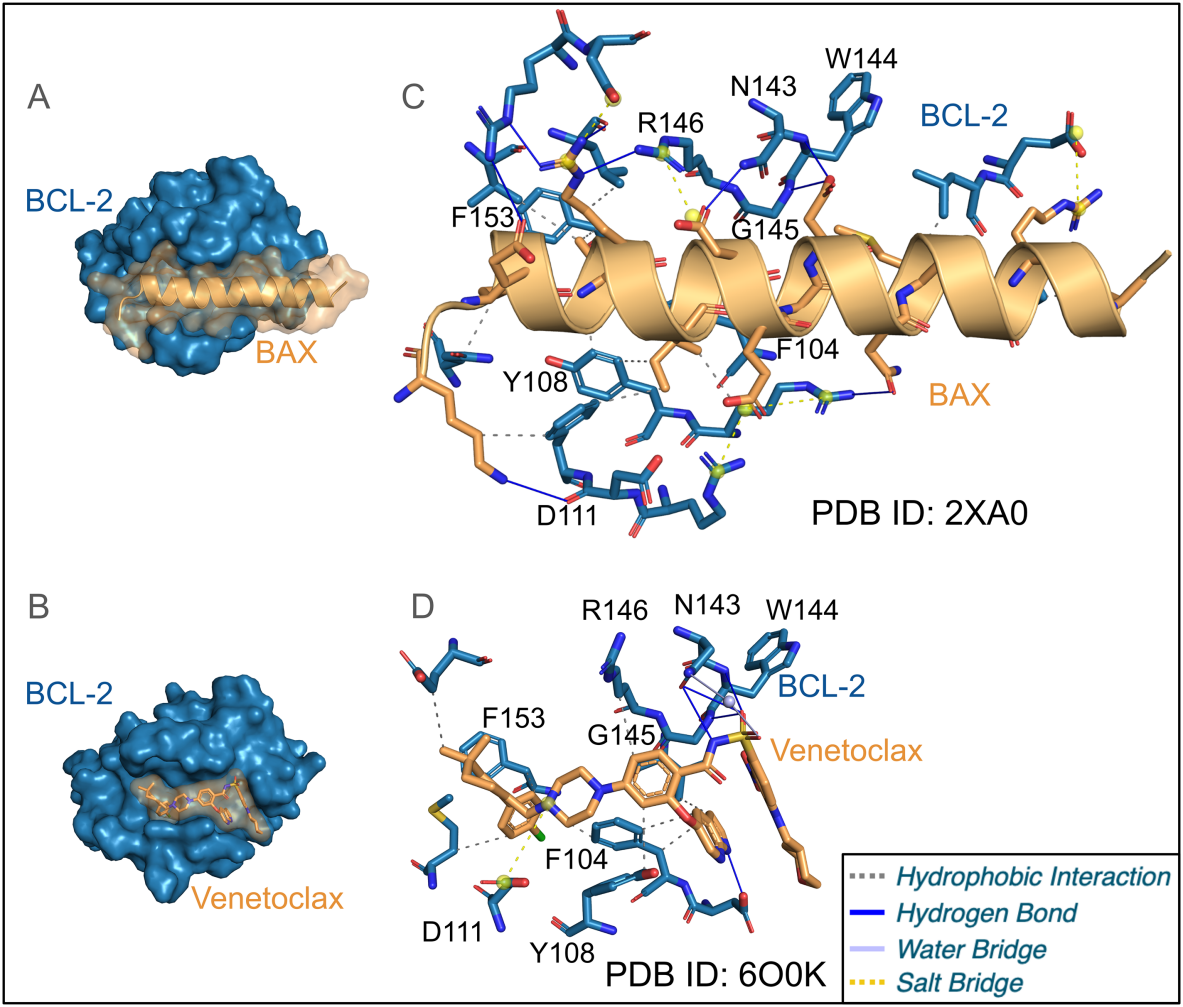
Bcl-2 interacting with the BAX protein and the small molecule venetoclax. **(A, B)** Surface view showing that BAX and venetoclax bind at the same binding site. **(C, D)** PLIP interactions for BAX/Bcl-2 and venetoclax reveal the key interacting residues of Bcl-2, a common hydrogen bond network of Bcl-2 residues N143, W144, and G145, and shared hydrophobic contacts with Bcl-2 residues F104, Y108, and F153.

Consider Fig. 1. PLIP reveals that BAX and venetoclax bind to Bcl-2 at the same interface [12, 14]. Concretely, PLIP shows that the Bcl-2 residues Phe104, Tyr108, Asp111, Asn143, Trp144, Gly145, Arg146, and Phe153 are common to both. BAX and venetoclax both bind to a hydrophobic groove formed by Phe104, Tyr108, and Phe153 via hydrophobic interactions. Moreover, they engage in a network of hydrogen bonds with Asn143, Trp144, and Gly145. Their binding to Bcl-2 is further supported by polar contacts with Asp111 and Arg146. The comparison of PLIP interaction patterns gives insights into the mechanism by which venetoclax mimics the interaction of Bcl-2 with BAX. The example illustrates how PLIP identifies key residues and interaction profiles in PLIs and PPIs, encouraging its use in structure-based drug discovery approaches.

## Using PLIP

The PLIP is freely available in three formats. The PLIP web server offers the most accessible option, allowing users to analyze PDB files by ID, upload custom PDB files, and adjust parameters such as distance thresholds for interaction detection within protein complexes. This flexibility makes the web interface ideal for characterizing individual structures.

For integration into custom analysis pipelines or high-throughput interaction analyses, the PLIP source code is available on GitHub. Users can locally implement PLIP via prepared containers (e.g. Docker or Singularity) or through manual installation, with detailed instructions provided.

As an intermediate solution between the intuitive web interface and local installation, we now offer a PLIP Jupyter notebook implementation, which can be run locally or on Google Colab. When run on Google Colab, the notebook features a graphical interface similar to the web server while maintaining the advantages of a local installation.

## Conclusion

PPIs are fundamental to many biological processes and are increasingly recognized as important targets in drug discovery, particularly for small molecules. In response to this, PLIP has been optimized to analyze PPIs, expanding its capabilities beyond PLIs. This enhancement allows for a detailed examination and visualization of non-covalent interactions within protein–protein complexes, essential for understanding binding mechanisms.

Additionally, the tool now offers users the flexibility to work across multiple platforms. Besides the traditional web interface and command line tool, PLIP is now available on Jupyter/Google Colab, providing an installation-free solution that can be easily customized for individual needs. This platform supports batch processing and allows for automated, Python-based evaluations, making it highly adaptable for larger workflows. These improvements are key to broadening the tool’s scope, particularly in the *in silico* drug design of small molecules targeting PPIs, offering researchers an advanced and user-friendly solution to accelerate their studies.

## Data Availability

The PLIP web server is online at https://plip-tool.biotec.tu-dresden.de.
